# Reed-Sternberg cells express CD161 and lectin-like transcript 1 in Hodgkin lymphoma

**DOI:** 10.3389/fmed.2026.1754727

**Published:** 2026-03-10

**Authors:** Anwar Rjoop, Rania Al-Samama'h, Laith Al-Eitan

**Affiliations:** 1 Department of Pathology and Microbiology, Faculty of Medicine, Jordan University of Science and Technology, Irbid, Jordan; 2 Department of Medical Laboratory Science, Faculty of Applied Medical Science, Jordan University of Science and Technology, Irbid, Jordan; 3 Department of Biotechnology and Genetic Engineering, Jordan University of Science and Technology, Irbid, Jordan

**Keywords:** CD161, FFPE, Hodgkin lymphoma, immunohistochemistry, LLT1, Reed-Sternberg cells, Immune Regulation, Immunotherapy Targets

## Abstract

**Introduction:**

Hodgkin lymphoma (HL) is a B-cell lymphoma, and it is diagnosed by the presence of Reed-Sternberg (RS) cells surrounded by heavy immune cell infiltration in a tissue biopsy. CD161 (Cluster of differentiation 161) and its ligand Lectin-like transcript 1 (LLT1) have recently emerged as a novel immune-regulatory axis that modulates natural killer (NK) and T cell-mediated function in cancer and inflammatory conditions, but their expression in RS cells and association with HL patients' clinical-pathological features remain poorly defined.

**Methods:**

In this study, 60 formalin-fixed paraffin-embedded (FFPE) tissue samples were collected from patients in Northern Jordan. In addition to 60 FFPE samples of positive control from benign reactive lymph node tissues and tonsils. Immunohistochemistry (IHC) was performed to assess the expression levels and patterns (cytoplasmic, membranous, or both) of CD161 and LLT1 in RS cells and then correlated with EBV status and clinical-pathological features such as subtype and disease stages.

**Results:**

LLT1 expression in RS cells was predominantly cytoplasmic, with occasional dual cytoplasmic and membranous expressions. This is the first study in the literature to detect CD161 expression in RS cells as neoplastic cells at protein level. Statistical analyses showed no significant association between LLT1 or CD161 expression in RS cells and HL subtype, stage or EBV status.

**Discussion:**

These findings provide the first characterization of LLT1 and CD161 expression in RS cells and suggest their potential use as a target in immunotherapy approaches in HL.

## 1 Introduction

Hodgkin lymphoma tumor microenvironment differs from other solid tumors by a high inflammatory milieu and a significantly low percentage of malignant cells. Reed-Sternberg (RS) cells encompass less than 1% of the total tumor microenvironment cell population (1, 2). HL subtypes are classified based on the difference in tumor microenvironment (TME) features (2–5). Classic HL (cHL) is characterized by mono-nucleated Hodgkin cells or binucleated RS cells, both of which are germinal center-derived malignantly transformed B-cells that express CD30, CD40, often CD15, and the transcriptional factor PAX5, but lose the expression of B-cell markers, such as CD45 and CD20 (3, 5–7). In contrast, nodular lymphocyte-predominant HL (NLPHL) is characterized by the presence of lymphocyte-predominant (LP) or popcorn cells (RS cell variant) that also originate from germinal-center transformed B-cells but retain the expression of B-cell markers, such as CD20, CD19, CD79a, CD45, and the transcriptional factors BOB1, PAX5, OCT2, and PU.1 (2, 5, 8, 9).

CD161 is the human ortholog of NKR-P1, and its ligand is LLT1 (lectin-like transcript 1), also known as CLEC2D (C-type lectin domain family 2, member D) protein (10–15). Both are C-type lectin-like receptors that may contribute to tumor immune escape upon binding (16, 17), and were found to be highly expressed in different types of cancers, including pancreatic, ovarian, breast, colon, lung, stomach, and other cancers (11, 16–28). However, their expression in Hodgkin lymphoma remains poorly studied (11, 26, 29–33). Therefore, this study investigates CD161 and LLT1 expression levels in RS cells and their association with patient clinical-pathological features, outcomes, and Epstein-Barr virus (EBV) infection.

Our findings demonstrated that LLT1 expression on RS cells was mainly cytoplasmic, with sporadic dual cytoplasmic and membranous expression. Notably, this is the first study to detect CD161 protein expression on RS cells as neoplastic cells. Although no significant associations were found between LLT1 or CD161 expression in RS cells and HL subtype, stage, or EBV status.

## 2 Materials and methods

### 2.1 Human samples and data collection

A total of 120 FFPE tissue samples were collected from King Abdullah University Hospital (KAUH) pathology department archives, which is the major hospital serving the north of Jordan. These FFPE samples include 21 patients with Nodular Sclerosis (NS) HL, 15 patients with Mixed Cellularity (MC) HL, 10 patients with Lymphocyte Rich (LR) HL, four patients with Lymphocyte Depleted (LD) HL, and six patients with Nodular Lymphocyte Predominant (NLP) HL. Four samples were excluded from the final analysis due to technical issues during sectioning or labeling, leaving 56 samples for full immunohistochemistry evaluation. In addition to 60 FFPE samples of positive control from benign reactive lymph node tissues and tonsils, used to assess the IHC staining protocols.

Patients' clinical-pathological features and diagnostic immunohistochemical data were collected and organized systematically (Table 1). Age at diagnosis, gender, HL subtype (classic HL vs. nodular lymphocyte-predominant HL), Ann Arbor staging at diagnosis (Stage I–IV), treatment regimen, follow-up outcomes (relapse, survival status, and time to recurrence), and the presence of extranodal involvement at diagnosis were obtained from medical e-records.

**Table 1:** Clinical and pathological characteristics of the study cohort.

**Parameters**	**Category**	**Count**	**Percentages (%)**
Population	Total cohort	56	100.0
Gender	Male	33	58.9
	Female	23	41.1
Patient HL subtypes	Nodular sclerosis	21	37.5
	Mixed cellularity	15	26.8
	Lymphocyte rich	10	17.9
	Lymphocyte depleted	4	7.1
	Nodular lymphocyte predominant	6	10.7
Stages	I	4	7.1
	II	16	28.6
	III	11	19.6
	IV	16	28.6
	N/A	9	16.1
Type of treatment	Chemotherapy	34	60.7
	Chemotherapy and radiation	8	14.3
	N/A	14	25.0
Experienced relapsed	No	29	51.8
	Yes	14	25.0
	N/A	13	23.2
Extranodal involvement	No	26	46.4
	Yes	14	25.0
	N/A	16	28.6
OS status	Alive	41	73.2
	Died	5	8.9
	N/A	10	17.9
EBV results	Positive	35	62.5
	Negative	21	37.5

### 2.2 Immunohistochemistry (IHC) procedure

Immunohistochemistry (IHC) staining was performed using the fully automated Ventana BenchMark ULTRA system (Ventana Medical Systems, Tucson, AZ, USA) at the pathology laboratory. First, the tissues were sectioned using a microtome at a 3–4 μm thickness, then the sections were mounted onto positively charged slides (Superfrost^®^ Plus) and incubated in an 87 °C drying oven for 30 minutes to facilitate and enhance tissue adherence. Next, the prepared slides were uploaded to the fully automated staining system. For deparaffinization and antigen retrieval, slides were automatically deparaffinized using the Ventana EZ Prep solution (catalogue No. 950–100, Ventana). Heat-induced epitope retrieval (HIER) was performed using CC1 (Cell Conditioning 1) buffer (Tris-EDTA-based, pH ~9.0; catalogue No. 950–124, Ventana) and heated within the instrument to expose antigen-binding sites. The primary antibody detection was performed using the OptiView DAB IHC Detection kit (Ventana), which employs a multimer-based secondary detection system and DAB (3,3'-diaminobenzidine) as the chromogen, creating a brown precipitate at the site of antigen expression. Hematoxylin II was used as a counterstain, and a bluing reagent was used to enhance nuclear contrast.

**Figure 1:**
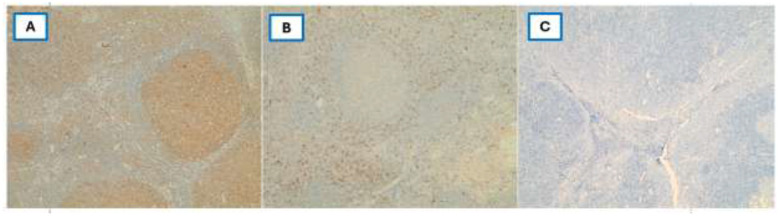
**(A–C)** Positive and negative controls of tonsillar tissue. **(A)** LLT1 expression showing membranous/cytoplasmic staining in lymphoid cells within tonsillar follicles. **(B)** CD161 expression highlighting scattered lymphocytes in the interfollicular areas. **(C)** Negative control by omission of the primary antibody showed complete absence of staining (IHC stain, 20x).

The sections were incubated with primary antibodies specific to CD161 (anti-KLRB1/CD161 mAb clone 3D8F5, Proteintech Catalog No. 67537-1-Ig, RRID: AB_2882756, 1:1,000), LLT1 (anti-CLEC2D mAb clone 4C7, Abnova, Catalog No. H00029121-M01, RRID: AB_463941, 1:50), CD8 (anti-CD8 mAb clone 4B11, MyBioSource Catalog No. MBS215242, RRID: AB_10887243, 1:50), and EBV (anti-EBV/LMP-1 mAb clone S20-D, MyBioSource Catalog No. MBS684063, 1:100). The positive controls (tonsillar tissue) were used to ensure the optimization of the staining procedure for CD161 and LLT1, and negative controls were used by omission of the primary antibody. Due to limited availability, isotype control antibodies were not performed; however, omission of the primary antibody showed complete absence of staining, and positive controls confirmed antibody specificity, (Figure 1).

Although isotope control antibodies were not used in this study, antibody validation relied on multiple established approaches, including 1) use of well-validated, commercially standardized antibodies previously used in peer-reviewed studies. 2) appropriate positive control tissue known to express CD161 and LLT1 3) expected membranous/cytoplasmic staining pattern and cellular localization consistent with published literature 4) negative controls with omission of the primary antibody and finally positive and negative internal controls.

For scoring CD161 and LLT1 expression in Reed-Sternberg cells, a combined intensity and percentage scoring system was used due to the limited number and uniform nature of RS cells. Staining intensity: 0 = negative, 1 = weak, 2 = moderate, and 3 = strong. The percentage of positive Reed–Sternberg (RS) cells was defined as the ratio of positively stained RS cells to the total number of RS cells examined. Ten high-power fields (HPFs) were analyzed for each case, and the average was calculated to ensure accuracy. Cells were classified as positive if they demonstrated membranous and/or cytoplasmic staining, independent of staining intensity. Cytoplasmic staining was defined as diffuse or granular intracellular staining confined to the cell body without circumferential outlining, whereas membranous staining was identified by linear or circumferential accentuation along the cell membrane, often in combination with cytoplasmic staining (dual pattern). Staining intensity was judged based on the control positivity intensity. CD30 was performed in parallel as a reference for the membranous expression patterns on RS cells. Then the final RS cell score was calculated by multiplying the intensity score by the percentage score to finally get a product between zero and 300, and then the expression levels were subcategorized according to the median; scores ≤ the median were classified as the “low expression” group, and scores > the median were classified as the “high expression” group. The scoring was performed independently by a pathology consultant (AR) and a trainee researcher (RA). Both were blinded to the clinical data, and any discrepancies were resolved through joint review using a multi-headed microscope.

### 2.3 Statistical analysis

Data analyses were performed using IBM SPSS Statistics version 27.0 (IBM Corp., Armonk, NY, USA). Inferential and descriptive statistical methods were utilized to assess the immunohistochemical expression patterns and their possible clinical significance. Normality of the IHC scores and the continuous variables was assessed using the Shapiro–Wilk tests and Q‘–Q plots, and all variables showed deviations from normality with statistically significant *p*-values < 0.05. Hence, non-parametric tests were used, including Kruskal–Wallis for comparisons between more than two groups, Mann–Whitney *U* for comparisons between two groups, and Spearman rho for relations between continuous variables. Overall survival (OS) and progression-free survival (PFS) were assessed by Kaplan–Meier survival analysis. Cases with incomplete follow-up dates (lost) or alive and did not experience the event (death or relapse) at the time of data collection were considered censored. This study is exploratory in nature, and the findings are interpreted in light of the small cohort size. Cases with missing or improper staining results were excluded from related statistical comparisons, and *p*-values < 0.05 (two-sided) were considered statistically significant.

### 2.4 Ethical considerations

Ethical approval for this study was obtained before the collection of samples and data from the Institutional Review Board at Jordan University of Science and Technology (JUST) and King Abdullah University Hospital (KAUH; IRB Approval Number: 2024/565). This study was conducted in accordance with the 1964 Helsinki Declaration and its later amendments. Informed consent was waived due to the retrospective and anonymized nature of the samples.

## 3 Results

### 3.1 LLT1 expression on Reed-Sternberg (RS) cells

**Figure 2:**
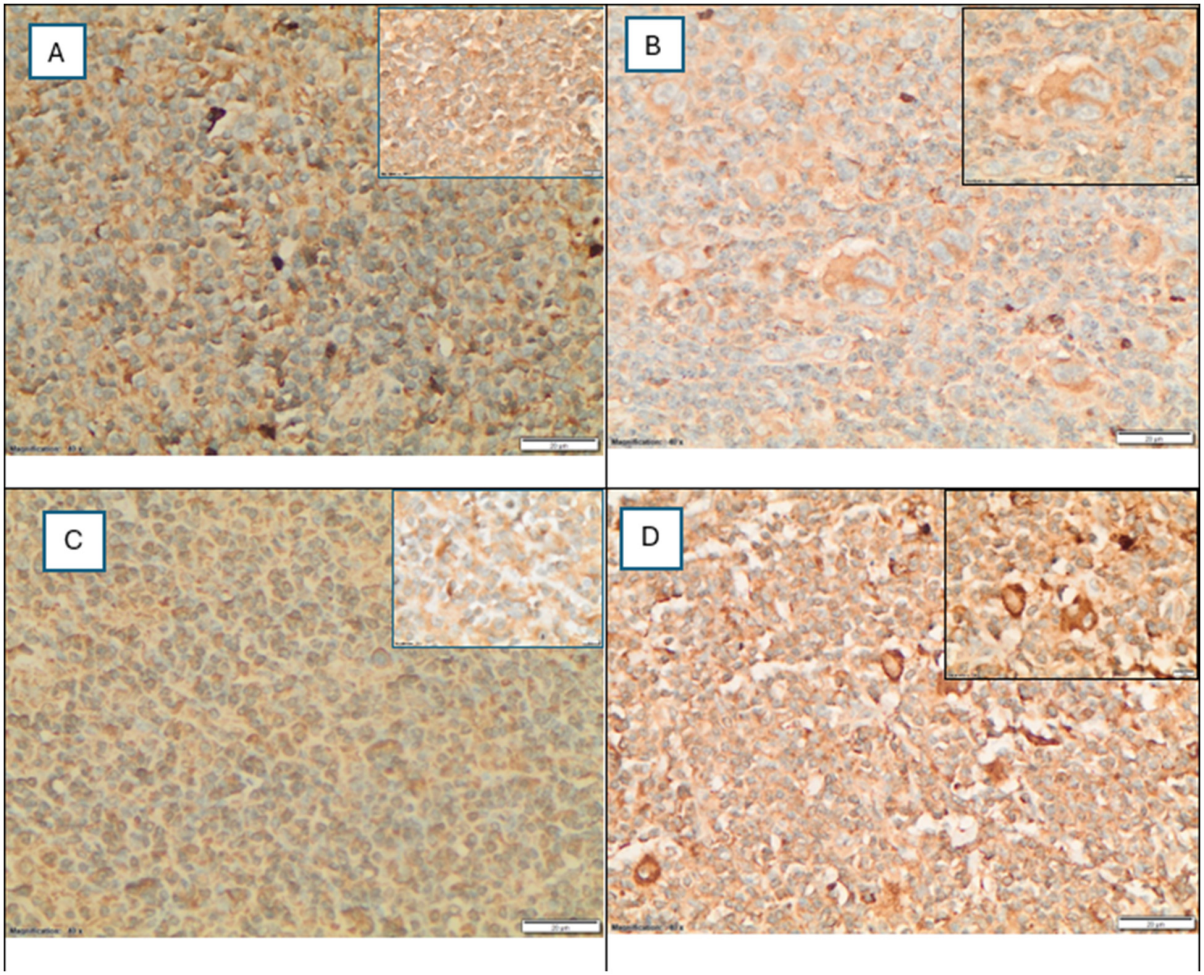
**(A–D)** LLT1 expression patterns in Reed–Sternberg RS cells by immunohistochemistry (40x and 100x inset). **(A)** A mixed pattern of negative and low-level cytoplasmic-only expression on RS cells. **(B)** High-level cytoplasmic-only expression. **(C)** Low-level dual cytoplasmic and membranous expression. **(D)** High-level dual cytoplasmic and membranous expression. The total expression level was calculated by multiplying the stain intensity score by the percentage of positive RS cells. Based on the median score, patients were classified into low expression (≤180; median), and high expression (>180; median) groups.

Assessing the expression of LLT1 in 56 HL tissues (Figure 2); LLT1 in RS cells was detected in all cases, and the expression was mainly cytoplasmic in 92.9% of the cases (52/56), and only four cases (7.1%) had both cytoplasmic and membranous expression of LLT1 on RS cells. HL cases that showed the dual LLT1 expression were two nodular sclerosis, one lymphocyte-rich, and one lymphocyte-depleted. No restricted membranous LLT1 expression on RS cells was detected in any case in our cohort.

### 3.2 CD161 expression on Reed-Sternberg (RS) cells

**Figure 3:**
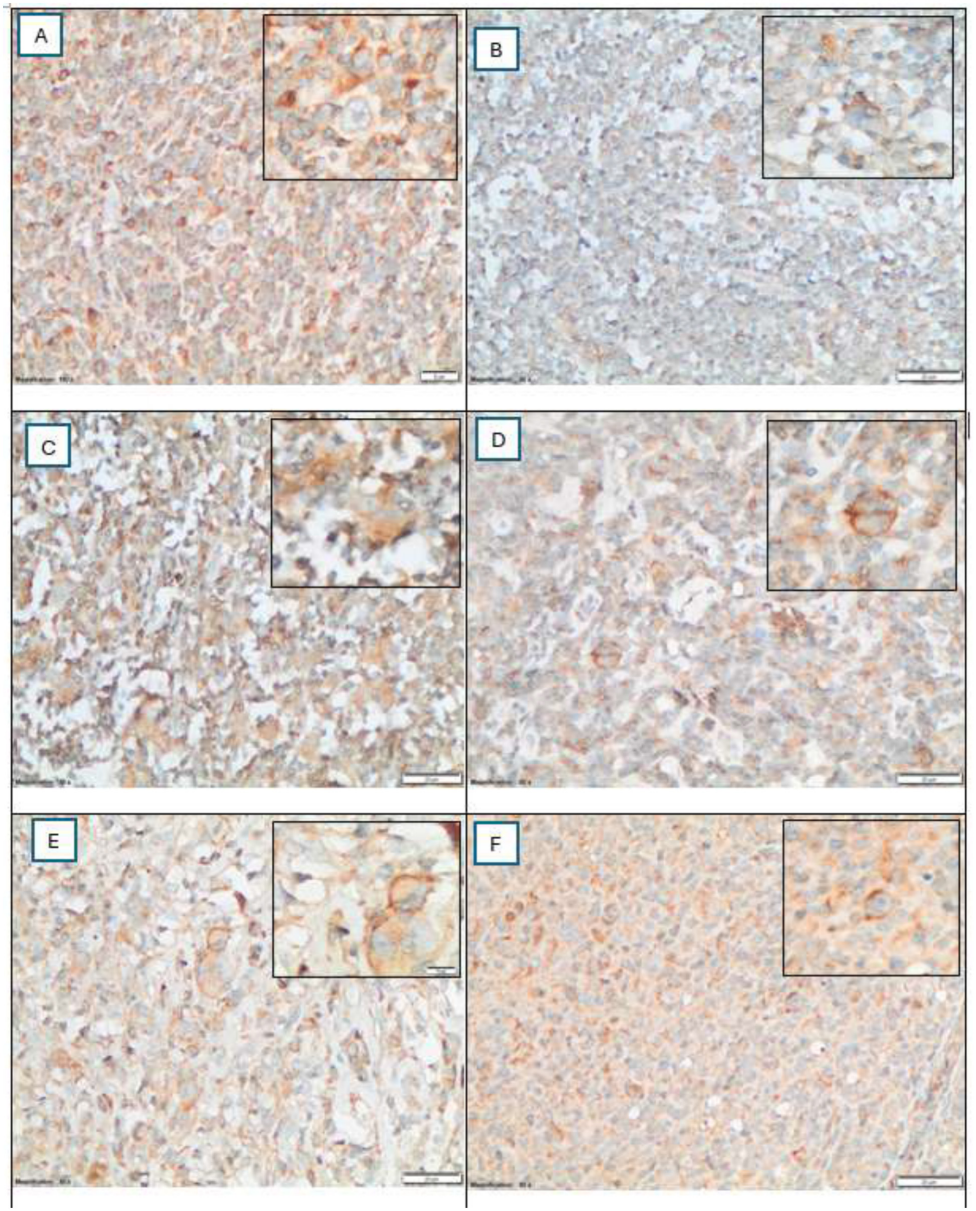
**(A–F)**. CD161 expression patterns in Reed–Sternberg RS cells by immunohistochemistry (40x and 100x inset). **(A)** No detectable CD161 expression in RS cells, background immune cells are positive. **(B)** Low-level cytoplasmic only expression. **(C)** High-level cytoplasmic only expression. **(D)** High-level membranous-only expression. **(E)** Low-level dual cytoplasmic and membranous expression. **(F)** High-level dual cytoplasmic and membranous expression. The total expression level was calculated by multiplying the stain intensity score by the percentage of positive RS cells. Based on the median score, patients were classified into low expression (≤90; median), and high expression (>90; median) groups.

Assessing the expression of CD161 in 56 HL tissues (Figure 3); CD161 expression was detected on RS cells, and it was mainly cytoplasmic in 44 cases (78.6%); three cases (5.4%) had membranous-only expression; four cases (7.1%) had cytoplasmic and membranous expression; and five cases (8.9%) had no CD161 expression. To our knowledge, this is the first study to report CD161 protein expression directly on RS cells in Hodgkin lymphoma.

### 3.3 LLT1 and CD161 expression levels on RS cells and EBV patients' status

Epstein–Barr virus (EBV) status was evaluated in all 56 HL cases in our cohort. Thirty five (62.5%) cases were EBV positive, and 21(37.5%) cases were negative, Table 1. Strong associations between EBV status and HL subtypes, such as mixed cellularity and lymphocyte-rich (Chi-Square test, *p* = 0.012). To investigate whether EBV infection could potentially contribute to differences in LLT1 and CD161 expression levels, the Mann–Whitney *U*-test was used to study the differences between EBV-positive and EBV-negative HL patients. Thus, there is no significant difference in LLT1 or CD161 expression levels on RS cells between EBV-positive and EBV-negative groups (*p* = 0.392 and *p* = 0.376, respectively; Tables 2, 3).

**Table 2:** Significance of LLT1 expression on RS cells and clinical-pathological features.

**Correlations of LLT1 expression on RSCs**	**Statistical Tests**	**Significance**	***p*-value**
HL subtypes	Independent-samples Kruskal–Wallis test	Not significant	0.354
HL stages	Independent-samples Kruskal–Wallis test	Not significant	0.131
Experiencing relapsed	Independent-samples Mann–Whitney *U*-test	Not significant	0.463
Extranodal involvement	Independent-samples Mann–Whitney *U*-test	Not significant	0.528
Gender	Independent-samples Mann–Whitney *U*-test	Not significant	0.649
CD161 expression in RSCs	Spearman ranks	Not significant	0.734
Age at diagnosis	Spearman ranks	Not significant	0.139
EBV	Independent-samples Mann–Whitney *U*-test	Not significant	0.392

**Table 3:** Significance of CD161 expression in RS cells and clinical-pathological features.

**Correlations of CD161 expression on RSCs**	**Statistical tests**	**Significance**	***p*-value**
HL subtypes	Independent-samples Kruskal–Wallis test	Not significant	0.256
HL stages	Independent-samples Kruskal–Wallis test	Not significant	0.806
Experiencing Relapsed	Independent-samples Mann–Whitney *U*-test	Not significant	0.419
Extranodal involvement	Independent-samples Mann–Whitney *U*-test	Not significant	0.705
Gender	Independent-samples Mann–Whitney *U*-test	Not significant	0.854
Age at diagnosis	Spearman ranks	Not significant	0.638
EBV	Independent-samples Mann–Whitney *U*-test	Not significant	0.376

### 3.4 Correlations of LLT1 and CD161 expressions in RS cells and patients' clinical-pathological features

Statistical analyses revealed no significant association between LLT1 expression levels in RS cells and the patients' clinical-pathological features, including HL subtype (Kruskal–Wallis, *p* = 0.354), clinical stage (Kruskal–Wallis, *p* = 0.131), relapse (Mann–Whitney U, *p* = 0.463), extranodal involvement (Mann–Whitney U, *p* = 0.528), or patient gender (Mann–Whitney U, *p* = 0.649). Likewise, LLT1 expression did not correlate significantly with patient age at diagnosis (Spearman, *p* = 0.139) or CD161 expression in RSCs (Spearman, *p* = 0.734, Table 2).

Additionally, statistical analyses demonstrated no significant associations between CD161 expression in RS cells and the patients' clinical-pathological features. CD161 expression did not differ across HL subtypes (Kruskal–Wallis test, *p* = 0.256) or disease stages (Kruskal–Wallis test, *p* = 0.806). Similarly, no significant differences were observed with relapse (Mann–Whitney U, *p* = 0.419), extranodal involvement (Mann–Whitney U, *p* = 0.705), or patients' gender (Mann–Whitney U, *p* = 0.854). Moreover, CD161 expression in RS cells showed no correlation with age at diagnosis, as indicated by Spearman's rank test (*p* = 0.638, Table 3).

### 3.5 LLT1 and CD161 expression in RS cells and HL patients' outcomes

#### 3.5.1 LLT1 and CD161 Expression in RS Cells and Overall Survival (OS)

**Figure 4:**
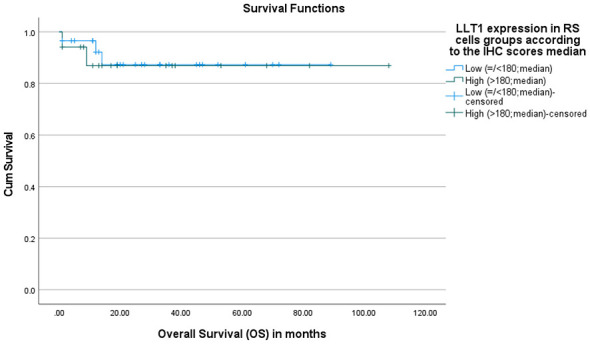
Overall survival (OS) according to LLT1 expression in RS cells. Kaplan–Meier curves OS stratified by LLT1 expression in RS cells based on immunohistochemistry score median. Patients were grouped into low LLT1 expression (≤180) and high LLT1 expression (>180). Tick marks indicate censored (alive/lost) cases. There was no statistically significant difference in overall survival between patients with low and high LLT1 expression in RS cells (*p* = 0.828).

The association between LLT1 and CD161 expression on Reed–Sternberg (RS) cells and Overall Survival (OS) was assessed by Kaplan-Meier survival analysis. There was no statistically significant difference in overall survival between patients with low and high LLT1 expression in RS cells (*p* = 0.828). Despite a slightly higher mean survival of 94 months in the high LLT1 group, this difference is not significant (Figure 4).

**Figure 5:**
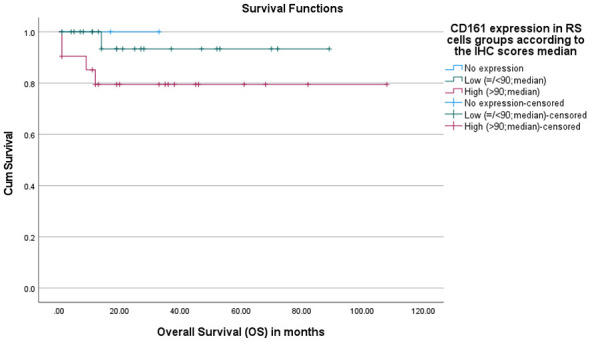
Overall survival (OS) according to CD161 expression in RS cells. Kaplan-Meier curves OS stratified by LLT1 expression in RS cells based on immunohistochemistry score median. Patients were grouped into low LLT1 expression (≤90) and high LLT1 expression (>90). Tick marks indicate censored (alive/lost) cases. No statistically significant difference in overall survival was observed among groups with different CD161 expression levels in RS cells (*p*-value = 0.277).

Furthermore, no statistically significant difference in overall survival was observed among groups with different CD161 expression levels in RS cells (*p*-value = 0.277). Mean and median survival times were not calculated because of the high proportion of censored cases (89.1%), and the curves for the “no expression” and “low expression” groups appear truncated due to the absence or low number of event numbers: 100% censored (alive/lost) in the “no expression” group and 95.7% in the “low expression” group (Figure 5).

#### 3.5.2 LLT1 and CD161 expression on RS cells and Progression-Free Survival (PFS)

**Figure 6:**
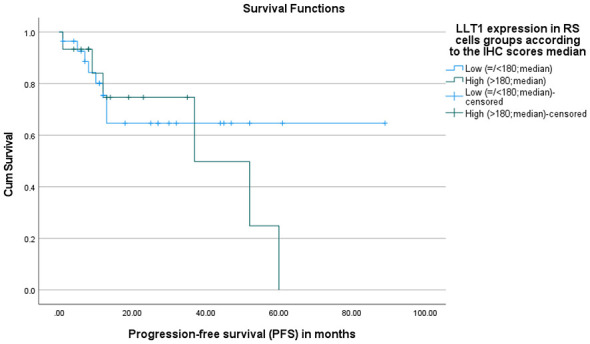
Progression-free survival (PFS) according to LLT1 expression in RS cells. Kaplan–Meier curves showing progression-free survival (PFS) stratified by LLT1 expression in Reed–Sternberg (RS) cells based on the median immunohistochemistry (IHC) score. Patients were categorized into low LLT1 expression (≤180) and high LLT1 expression (>180). Tick marks indicate censored (alive/lost) cases. No statistically significant difference in progression-free survival between patients with low and high LLT1 expression on RS cells (*p* = 0.525).

The association between LLT1 and CD161 expression in RS cells and progression-free survival (PFS) was assessed by Kaplan-Meier survival analysis. No statistically significant difference in progression-free survival between patients with low and high LLT1 expression on RS cells (*p* = 0.525). Although the low LLT1 expression group shows a higher mean PFS of 60 months, while the higher group has 39 months, this difference is not statistically significant in our cohort (Figure 6).

**Figure 7:**
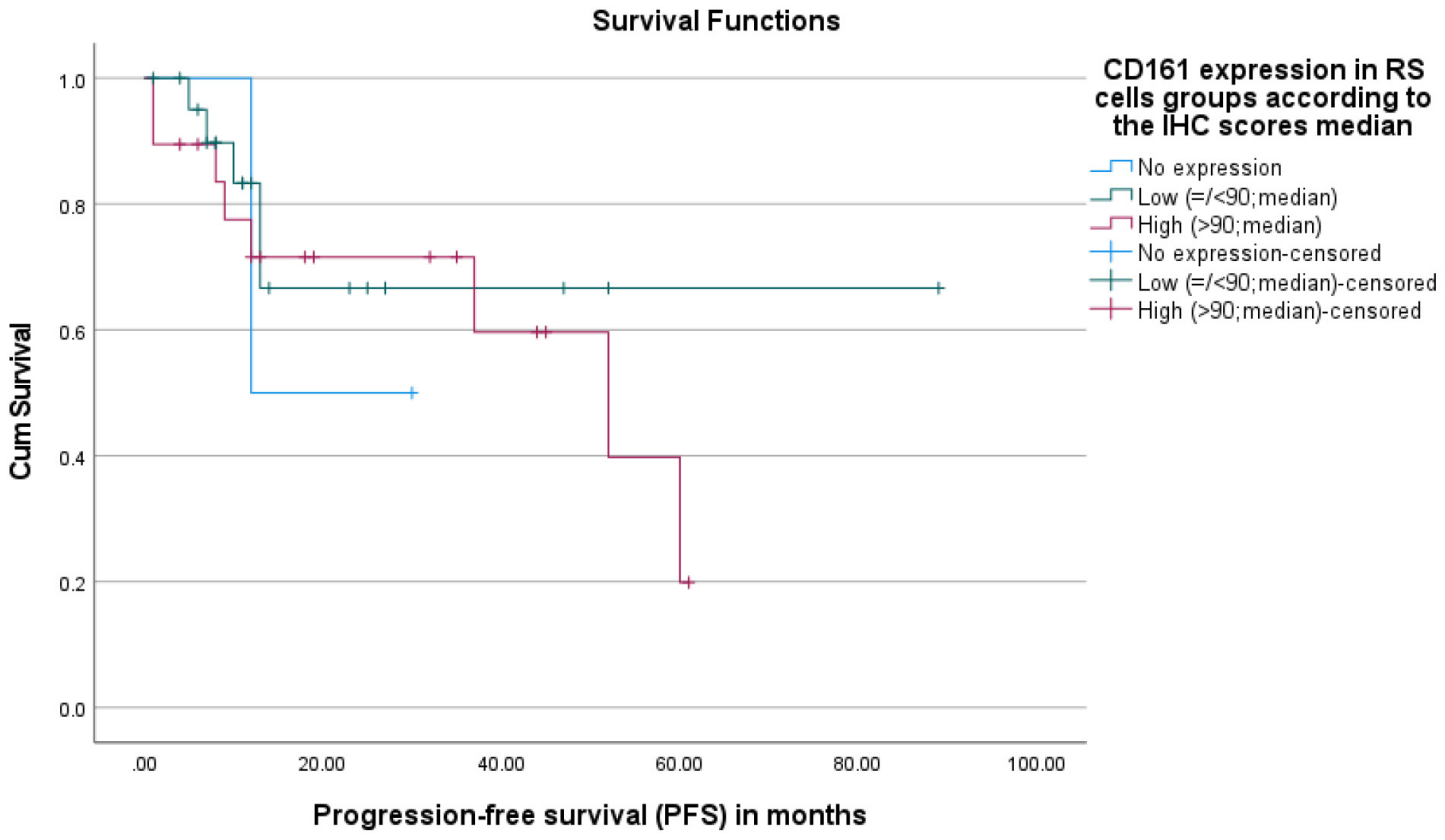
Progression-free survival (PFS) according to CD161 expression in RS cells. Kaplan–Meier curves showing progression-free survival (PFS) stratified by LLT1 expression in Reed–Sternberg (RS) cells based on the median immunohistochemistry (IHC) score. Patients were categorized into low LLT1 expression (≤90) and high LLT1 expression (>90). Tick marks indicate censored (alive/lost) cases. No statistically significant difference in progression-free survival between patients with low and high LLT1 expression on RS cells (*p* = 0.734).

For CD161, one case from the “no expression” group had a progression event (relapsed/death), five cases from the low-expression group, and eight cases from the high-expression group had a progression event (relapsed/death). The median PFS for the high-expression group was 52 months, whereas the median was not reached for the low-expression group due to a high proportion of censored (alive/lost) cases. The no-expression group had a median PFS of 12 months. There was no statistically significant difference in PFS among the three groups (*p* = 0.734; Figure 7).

## 4 Discussion

The interaction between CD161 (KLRB1) and its ligand, lectin-like transcript 1 (LLT1), has recently emerged as a novel immune-regulatory axis (21, 24, 25, 34). Their expression affects the behavior of cancer and immune system cells, which highlights the potential to use them in immunotherapy. In glioma cells, blocking CD161/LLT1 interaction with anti-CD161 monoclonal antibodies increased T cell-mediated cytotoxicity against tumor cells (17). Bernstein et al. used high-affinity CD161 antibodies to inhibit the CD161/LLT1 ligation in GC-derived B-cell lymphomas, which led to enhanced NK and T-cell proliferation, increased tumor infiltration, and cytotoxicity against cancerous B cells (26). Despite the growing knowledge of CD161 and LLT1's role in chronic malignancies and inflammatory conditions (16, 20, 24, 25, 35). Their expression patterns in HL are still poorly studied.

In the study cohort, LLT1 expression was detected in RS cells, primarily in the cytoplasm, with only four cases exhibiting both cytoplasmic and membranous LLT1 expression. All four cases were classic Hodgkin lymphoma, including two cases of nodular sclerosis, one case of lymphocyte-rich, and one case of lymphocyte-depleted. In NLPHL cases, LLT1 expression in neoplastic lymphocyte-predominant (LP) or popcorn cells was exclusively cytoplasmic. However, Llibre et al. demonstrated stronger LLT1 expression in NLPHL compared to the weak expression in cHL (30). While Germain et al. demonstrated weak positive LLT1 expression in only two of 23 cHL cases (29). These studies did not systematically characterize the LLT1 expression pattern or location and focused on neoplastic B cells in non-Hodgkin lymphomas (NHLs), offering limited insight into LLT1 in HL. In addition to IHC-based studies, Alvarez Calderon et al. employed mRNA database analyses to investigate CLEC2D/LLT1 expression among cancer types, revealing high CLEC2D levels in hematological malignancies, including HL (31). CLEC2D has three isoforms, including the LLT1 (isoform 1), and it is the surface functional ligand for the CD161 receptor. While isoforms two and four are intracellular and result from alternative splicing and are localized within the endoplasmic reticulum, the exact biological roles of isoforms two and four are not well defined (11). While the membranous LLT1 mediates immune evasion through interaction with CD161, cytoplasmic LLT1 likely reflects different stages of intracellular synthesis, trafficking, or storage, so cytoplasmic expression may indicate an active LLT1 regulatory axis and potential functional immune modulation. Although this data suggests LLT1 is especially relevant in lymphoid cancers, it does not provide information on localization nor differentiate between malignant cells and immune populations within the HL microenvironment. Our findings in HL detail LLT1/CLEC2D expression in malignant RS cells, including locations and percentages of positive cells, providing a broader and more detailed evaluation of LLT1. The detection of LLT1 in this study may also reflect differences in methodological approaches, including the use of different antibody clones, detection sensitivity, and scoring criteria.

Notably, CD161 expression was observed in RS cells; 44 cases exhibited cytoplasmic-only expression, three exhibited membranous-only expression, four cases exhibited dual expression, and five cases had no expression. Our study is the first to detect CD161 protein expression in RS cells in HL, not just in background inflammatory cells (BICs). This observation aligns with findings from Li et al., who used single-cell sequencing (scRNA-seq) and analyzed CD161 expression across individual cells in several tumors, like lung, pancreatic, prostate adenocarcinomas, and others, and found that CD161 expression was not NK and T-cell restricted, but also tumor and stromal cells may exhibit CD161 expression (19). Together with our results, this may suggest that CD161 expression can be induced on tumor cells within the TME, potentially contributing to tumor-immune interactions, immune evasion, or blending with surrounding immune cells by phenotypically mimicking them.

LLT1 and CD161 expressions in RS cells were not influenced by EBV status. Germain et al. reported that EBV could directly induce LLT1 on infected B cells. Thus, EBV infection of peripheral B cells and tonsillar B cells *in vitro* and *in vivo* resulted in upregulation of LLT1 expression (36). Bernstein et al., LLT1 IHC results demonstrated that both EBV-positive and EBV-negative HL cases exhibited LLT1 expression regardless of EBV status (26).

In fact, RS cells were primarily B cells before transforming into malignant cells and may exhibit a heterogeneous LLT1 expression pattern regardless of EBV status (2, 5). As is known, EBV is strongly associated with cHL occurrence, most commonly in the mixed cellularity subtype (37, 38), as our data also confirmed (*p*-value = 0.012). The EBV effect in the HL tumor microenvironment is more complex, potentially involving regulatory pathways, cytokine profiles, or immune evasion mechanisms.

Similarly, there was no significant association with HL subtypes, HL stage, extranodal involvement, patient age, patients' OS, or PFS. These insignificant findings are still noteworthy because they may suggest that the expression of LLT1 and CD161 may be stable in most clinical-pathological features and may indicate their expression patterns are primarily determined by local microenvironment factors rather than clinical features.

## 5 Conclusion

Despite the small sample size, especially within certain subgroups, like lymphocyte-depleted and nodular lymphocyte-predominant HL subtypes and the lack of using isotype controls. Our retrospective, cross-sectional design study gives a novel insight regarding LLT1 and CD161 expression patterns. It opens the door for more studies to further investigate the expression changes in different HL patient cohorts, including relapsed/refractory classical Hodgkin lymphoma, patients with poor response to PD-1 blockade and advanced-stage or high-risk HL (IPS ≥3). Other methods that might be used to establish therapeutic significance also include comparing the difference in LLT1 and CD161 expression between circulating and tumor-infiltrating immune cells by collecting tumor tissues paired with peripheral blood samples and analyzing the samples by multiplex immunophenotyping/spatial analysis. Furthermore, validate the LLT1/CD161 function of interaction in HL TME by functional assays such as functional NK/T-cell cytotoxicity and cytokine assays. Antibody blockade experiments targeting LLT1/CD161 to assess the anti-tumor immune activity. Additionally, investigating their expression patterns in other lymphoid malignancies, such as diffuse large B-cell lymphoma and chronic lymphocytic leukemia, may clarify whether this immune-evasion mechanism is shared across related tumors. In conclusion, both LLT1 and CD161 are upregulated in HL. Thus, position this LLT1/CD161 axis as a potential target in immunotherapy approaches to treat HL.

## Data Availability

The raw data supporting the conclusions of this article will be made available by the authors, without undue reservation.
